# MULTISYSTEM INFLAMMATORY SYNDROME IN A CHILD ASSOCIATED WITH CORONAVIRUS DISEASE 19 IN THE BRAZILIAN AMAZON: FATAL OUTCOME IN AN INFANT

**DOI:** 10.1590/1984-0462/2020/38/2020165

**Published:** 2020-08-26

**Authors:** Emmerson Carlos Franco de Farias, Maria Cleonice Aguiar Justino, Mary Lucy Ferraz Maia Fiuza de Mello

**Affiliations:** a Hospital Fundação Santa Casa de Misericórdia do Pará, Belém, PA, Brazil.; b Instituto Evandro Chagas, Ananindeua, PA, Brazil.

**Keywords:** COVID-19, Multiple organ failure, Intensive care units, Pediatric, COVID-19, Insuficiência de múltiplos órgãos, Unidades de terapia intensiva pediátrica

## Abstract

**Objective:**

Recently, there have been reports of children with severe inflammatory syndrome and multiorgan dysfunction associated with elevated inflammatory markers. These cases are reported as presenting the Multisystem Inflammatory Syndrome in Children (MIS-C) associated with COVID-19. In this study, we describe with parental permission a case of MIS-C in an infant with severe acute respiratory syndrome coronavirus 2 (SARS-CoV-2) infection.

**Case description:**

A seven-month-old infant, with SARS-CoV-2 infection and a history of extreme preterm birth and very low weight at birth, with an initial course of mild respiratory symptoms and abrupt progression to vasoplegic shock, myocarditis and hyperinflammation syndrome, shown by high levels of troponin I, ferritin, CRP, D-dimer and hypoalbuminemia. Despite the intensive care provided, the child developed multiple organ dysfunction and died.

**Comments:**

Patients with a history of extreme prematurity may present with MIS-C in the presence of COVID-19 and are a group of special concern.

## RESUMO

**Objetivo:**

Recentemente, foram descritos relatos de crianças com exame positivo para o coronavírus da síndrome respiratória aguda grave 2 (SARS-CoV-2) associado à disfunção de múltiplos órgãos, secundária à hiperinflamação, denominada de síndrome inflamatória multissistêmica pediátrica (do inglês *multisystem inflammatory syndrome in children -* MIS-C). O objetivo deste relato é descrever um caso de MIS-C em lactente com infecção por SARS-CoV-2 e com evolução fatal abrupta, a despeito do suporte de terapia intensiva pediátrica.

**Descrição do caso:**

Lactente de sete meses, com infecção por SARS-CoV-2 e antecedentes de prematuridade extrema, com quadro inicial de síndrome gripal e progressão abrupta para choque vasoplégico, miocardite e síndrome de hiperinflamação, evidenciados por níveis elevados de troponina I, ferritina, proteína C reativa (PCR), dímero D e hipoalbuminemia. Não obstante o suporte de terapia intensiva instituído, a criança evoluiu com disfunção de múltiplos órgãos e morte.

**Comentários:**

Pacientes com antecedentes de prematuridade extrema podem apresentar MIS-C na vigência de doença do coronavírus 19 (COVID-19) e constituir um grupo de preocupação especial.

## INTRODUCTION

The pandemic caused by the coronavirus disease 2019 (COVID-19) has affected millions of people around the world.^1^ The pediatric population seems to be much less affected than adults. A recent systematic review of the literature suggests^2^ that children represent less than 5% of the diagnosed cases of COVID-19, and usually present with milder forms of the disease. However, preschoolers, and especially infants, are vulnerable to the infection of severe acute respiratory syndrome coronavirus 2 (SARS-CoV-2), and children aged under one year would be more prone to developing severe or critical forms of the disease, with frequency^3^ of 10.1%.

Recently, there have been reports^4^ ^,^ ^5^ ^,^ ^6^ ^,^ ^7^ of children who were previously healthy and tested positive for SARS-CoV-2, progressing to severe inflammatory syndrome and presenting with characteristics that are similar to those of Kawasaki disease or toxic shock syndrome. They present with persistent fever and multiorgan dysfunction associated with high inflammatory markers. This case cluster was the base for the description of multisystem inflammatory syndrome in children - MIS-C), associated with COVID-19.

The diagnosis of MIS-C should be considered among children and adolescents aged from zero to 19 years, with characteristics of typical or atypical Kawasaki disease or shock syndrome, according to the case definition proposed by the World Health Organization (WHO)^8^, described in Chart 1.

**Chart 1:** Case definition^8^ of multisystem inflammatory syndrome according to the World Health Organization*.

1. Children and adolescents aged from zero to 19 years, with fever >3 days
And two of the following: Exanthema or bilateral non-purulent conjunctivitis or signs of mucocutaneous inflammation (oral, hands or feet). Hypotension or shock. Characteristics of myocardial dysfunction, pericarditis, valvulitis or coronary abnormalities (including echocardiographic findings), or elevated levels of troponin, NT-proBNP. Evidence of coagulopathy (by PT, APTT, high D-dimers). Acute gastrointestinal problems (diarrhea, vomit or abdominal pain)
And high inflammation markers: BSR, CRP or procalcitonin.
And no other obvious microbial cause for inflammation: Bacterial sepsis, staphylococcal or streptococcal shock syndromes.
And evidence or possible contact with patients with COVID-19: RT-PCR, positive antigen or serological test.

*NT-proBNP: terminal fragment of the b-type natriuretic peptide; PT: prothrombin time; APTT: activated partial thromboplastin time; BSR: blood sedimentation rate; CRP: C-reactive protein; RT-PCR: *reverse-transcriptase polymerase chain reaction*; COVID-19: coronavirus disease 19.

Currently, there is limited information about risk factors, pathogenesis, clinical course and treatment of MIS-C. In Brazil, there have not been reports of that syndrome, after an analysis of published cases about the subject. Therefore, it is relevant to register suspected cases in order to characterize this condition, which was recently recognized in the pediatric population, thus increasing the chances for health professionals to recognize it.

In this study, we describe a case of MIS-C in an infant infected with SARS-CoV-2, after parental authorization, which had a fatal outcome despite the support received in pediatric intensive care.

## CASE REPORT

Female infant, aged seven months, brown, weighting 4.5 kg, 57 cm high, born of natural birth, with 26 weeks and two days of gestational age, in a reference hospital in Belém, Pará, Brazil. After birth, she was referred to a neonatal intensive care unit (NICU) in the same service, and remained hospitalized for four months due to acute respiratory distress symptom (ARDS) and late neonatal sepsis.

After this period, she was transferred to the neonatal intermediate care unit, and stayed there for 45 days with prematurity chronic lung disease and swallowing coordination problems, with indication for surgical gastrostomy (GTM) with fundoplication; however, she was hemodynamically stable, with no need for antibiotic therapy. During this period, transfontanellar ultrasound was performed and showed periventricular hemorrhage restricted to the general matrix, and echocardiography showed foramen ovale pervium (diameter of the atrial derivation smaller than 2mm, and minimum hemodynamic impact), with 44 weeks of corrected age. Vaccines were up to date, and the infant also received palivizumab, according to the age group.

After surgical procedure (GTM), she was transferred to the pediatric nursery for care and diet progression. On the fifth post-operative (PO) day, she was stable, GTM had good aspect and diet was full. The perspective was for hospital discharge, and the family would be trained to posteriorly use the homecare service.

On the seventh PO, day 1 (D1), she presented with high fever, between 38 and 38.5°C, alternated irritability and periods of lethargy, dry cough, decreased oxygen saturation (O_2_ Sat), up to 85% during the coughing attacks, and watery diarrhea (four episodes). Oxygen therapy was implemented with a nasal catheter (5 L/min), with improved O_2_Sat (97%). Initial lab tests did not show major changes (Tables 1, 2, 3 and 4).

**Table 1:** Results of the blood cell count and renal function, during the hospitalization period due to the coronavirus disease 2019.

Lab tests	D1	D3	D4	Reference range
Hemoglobin	11	11.4	13.9	12-18 g/dL
Hematocrit	32.5	35	40	36-55 %
Red blood cells	4.01	4.04	4.69	3.9-6.7/mm^3^
MCV*	80	86.7	85.4	80-100 fl
MCH*	27.2	28.3	29.7	25-35 pg
RDW*	12.2	13.9	17.5	11.6-15.9 %
Total leukocyte count/mm^3^	13,950	22.990	12,160	4,000-10,000/mm^3^
Band cells/mm^3^ (%)	279 (2%)	459 (2%)	0 (0%)	0-200/mm^3^
Segmented/mm^3^ (%)	7,533 (54%)	15,771 (68%)	6,809 (56%)	1,500-6,000/mm^3^
Lymphocytes/mm^3^ (%)	5,162 (37%)	5,336 (23%)	770 (6.4%)	1,500-4,000/mm^3^
Monocytes/mm^3^ (%)	697 (5%)	1,333 (6%)	4,403 (36.2%)	400-1,000/mm^3^
Eosinophils /mm^3^ (%)	279 (2%)	0 (0%)	85 (0.7%)	40-400/mm^3^
Platelets/mm^3^	360,200	373,600	154,800	150-450,000/mm^3^
Urea	21	88	116	16-40 mg/dL
Creatinine	0.2	0.7	0.7	0.2-1.2 mg/dL
GFR* (mean/SD)	118	34	34	(96±22 mL/min/1.73 m^2^)

*D: day; MCV: mean corpuscular volume; MCH: mean corpuscular hemoglobin; RDW: red cell distribution width; GFR: glomerular filtration rate, adapted from Staples et al.^9^; SD: standard deviation. All tests were performed according to the protocols described by the manufacturers. It was chosen to define reference ranges according to age and sex.^10^ ^,^ ^11^

**Table 2:** Ionogram results and blood coagulation test during the period of hospitalization due to the coronavirus disease 2019.

Lab tests	D1	D3	D4	Reference range
Sodium	132	171	161	135-145 mmol/L
Potassium	3.6	5.3	6.8	3.5-5.5 mmol/L
Total calcium	9.0	5.1	7.7	8.5-0.2 mg/dL
Magnesium	2.3	2.8	1.9	1.7-2.6 mg/dL (0.7-1.1 mmol/L)
Chlorides	102	132	104	98-107 mmol/L
Phosphorus	3.1	1.2	1.1	2.5-4.5 mg/dL
CRP*	0.5	5.8	75.4	<0.6 mg/dL
BSR*	12	19	35	Up to 20 mm in the 1st hour
Ferritin	--	1,395	7,791	10-500 ng/mL
LDH*	--	--	1,291	115-25 UI/L
Uric acid	--	--	2.1	1.1-5.8 mg/dL
PT*	11.6	--	15.7	10-14”
APTT*	39.2	--	42.5	24-40”
INR*	1.01	--	1.48	0.8-1
D-Dimer	--	--	1,233	< 500 ng/dL
Fibrinogen	--	---	22.0	100-400 mg/dL

*D: day; CRP: C-reactive protein; BSR: blood sedimentation rate; LDH: lactate dehydrogenase; PT: prothrombin time; APTT: activated partial thromboplastin time; INR: international normalized ratio. All tests were performed according to the protocols described by the manufacturers. It was chosen to define reference ranges according to age and sex.^10^ ^,^ ^11^

**Table 3:** Results of liver, pancreatic function and myocardial injury, during the period of hospitalization due to coronavirus disease 2019.

Lab tests	D1	D3	D4	Reference range
Amylase	--	28	---	20-160 U/L
Lipase	---	32	---	<50 U/L
triglycerides	--	165	--	<100 mg/dL
Total cholesterol	--	180	--	<170 mg/dL
Troponin I	--	1,212	2,228	<2.0 ng/L
CPK*	--	--	165.4	30-300 U/L
CK-MB*	--	--	243	<25 U/L
AST*	42	44	98	<40-42 IU/L
ALT*	22	48	55	<42 IU/L
TB*	--	0.27	--	0.2-1.3 mg/dL
DB*	--	0.2	--	≤0.4 mg/dL
IB*	--	0.07	--	Up to 1.1 mg/dL
Albumin	3.5	3.0	2.3	3.5-4.7 g/dL
Lactate	1.0	3.4	7.0	0.5-2.0 mmol/L
Serum glycemia	78	301	77	60-100 mg/dL
Calculated serum osmolality	272	373	345	285-300 mOsm/kgH_2_O

*D: day; CPK: creatine phosphokinase; CK-MB: creatine kinase MB; AST: aspartate transaminase; ALT: alanine transaminase; TB: total bilirubin levels; DB: direct bilirubin levels; IB: indirect bilirubin. All tests were performed according to the protocols described by the manufacturers. It was chosen to define reference ranges according to age and sex.^10^ ^,^ ^11^

**Table 4:** Results of the arterial gasometry and central venous analyses during the period of hospitalization due to the coronavirus disease 2019.

Arterial gasometry	D1	D3	D4	Reference range
pH*	7.51	7.23	7,396	7.35-7.45
pCO_2_*	38.7	46.4	45.8	35-45 mmHg
pO_2_*	141	75	144	75-100 mmHg
BIC*	22.9	19.1	27.5	20-24 mmol/L
BE*	0.1	-7.7	2.6	-2/+2 mmol/L
O_2_Sat*	98.9	95	99.1	92-99%
AG*	11.90	28.80	16.70	8-16 mmol/L
SIDa*	43.90	48.80	66.40	38-42 mmol/L
SIDe*	37.60	22.40	30.70	38-42 mmol/L
SIG*	6.3	26.40	35.7	0
*PaO*_*2*_ */FiO*_*2*_ *	564	185	180	<300
OI*	--	6.4	8.8	4-8: mild; 8-16: moderate; >16: severe^α^
SvcO_2_*	--	56.5	79.5	65%

*D: day; pH: potential for hydrogen; pCO_2_: partial pressure of carbon dioxide; pO_2_: partial pressure of oxygen; BIC: sodium bicarbonate; BE: base excess; O_2_Sat: oxygen saturation; AG: anion gap; SIDa: strong ion difference apparent; SIDe: strong ion difference effective; SIG: strong ion gap; PaO_2_/FiO_2_: relation between arterial oxygen pressure and fraction of inspired oxygen; OI: oxygenation index; SvcO_2_: central venous oxygen saturation. SIDa was calculated by [sodium+potassium+magnesium+calcium]-[chloride+lactate]. SIDe was calculated by SIDe=[2.46×10^-8^×PaCO_2_ (mmHg)/10^-pH^+(albumin (g/dL)×(0.123×pH - 0.631)+(phosphate (mg/dL)×(0.309×pH - 0.469)]. SIG was calculated by the difference between SIDa and SIDe. The Anion Gap was calcualted by [Na]+[K]-[Cl+BIC].^12^The oxygenation index^13^ was calculated by FiO_2_×Mean Airway Pressure (MAP)×100/PaO_2_. On the first day (D1), in a nasal catheter with 0.5 L/min; on the third day (D3), FiO_2_=40% and MAP=12; on the fourth day (D4), FiO_2_=80% and MAP=16. ^α^The classification of acute respiratory distress symptom (ARDS) was based on the current pediatric criteria, according to the Pediatric Acute Lung Injury Consensus Conference (PALICC), 2015.^13^ All tests were performed according to the protocols described by the manufacturers. It was chosen to define reference ranges according to age and sex.^10^ ^,^ ^11^

**Figure 1:**
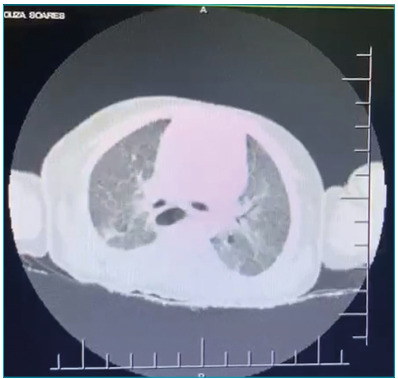
Axial images of the thoracic computed tomography, performed in the beginning of the hospitalization period, caused by the coronavirus disease 2019, showing bilateral ground-glass opacities in the mid an lower segments.

Thoracic computed tomography (CT) was requested and showed multiple confluent acinar opacities, with formation of areas of consolidation and air bronchogram, associated with diffuse ground-glass opacities, especially in posterior segments of the upper and basal lobes, bilaterally (Figure 1). Such findings had been present in a previous CT, with corrected age of 35 weeks, however, in lower proportion and compatible with chronic lung disease. Cranial CT had no changes.

With the suspicion of COVID-19, oropharyngeal aspiration was used, as well as reverse transcriptase-polymerase chain reaction (RT-PCR) tests for the SARS-CoV-2 virus, influenza A and B, metapneumovirus, adenovirus, parainfluenza types 1, 2, 3 and 4, respiratory syncytial virus (RSV). The result was positive only for SARS-CoV-2, and the test was performed in Laboratório Central do Estado (Lacen, state of Pará). According to the hospital’s protocol, cefepime, azithromycin and oseltamivir were administered. There was no bacterial growth in the cultures (stool, blood and urine). Serology for the Epstein-Barr virus, cytomegalovirus, toxoplasmosis, rubella, viral hepatitis (A, B, and C) and parvovirus B19 did not reveal recent infections.

On day 3 (D3), the patient became worse and presented a hypoxic crisis associated with lethargy, apnea, tonic-clonic seizure and signs of circulatory shock. She was transferred to the Pediatric ICU (PICU) after the installation of advanced life support. At the physical admission test in the PICU, the infant presented with continuous high fever (T: 39.1°C), without recrudescence with antipyretics (dipyrone and paracetamol). She showed minor reaction to manipulation, with sinus tachycardia (heart rate [HR]: 210 bpm), perioral cyanosis, paleness, mottled skin, peripheral capillary perfusion <2 seconds, and systemic blood pressure (SBP) of 61×25 mmHg.

The patient was submitted to mechanical ventilation (MV), in the assist-control mode, with pressured-controlled ventilation (PCV A/C), fraction of inspired oxygen (FiO_2_) of 40%, inspiratory pressure of 14 cmH_2_O, positive end-expiratory pressure (PEEP) of 7 cmH_2_O, respiratory rate of 25 and inspiratory time of 0.65 seconds. Ther was pesence of biliary output with brownish clots in the nasogastric tube and diuresis, however, reduced (1.2 mL/kg/h and glomerular filtration rate^9^ [GFR] of 33 mL/min/1.73 m^2^).

A central venous catheter was inserted in the right subclavian vein for the infusion of fluids and vasoactive drugs. In the laboratory analysis^10^ ^,^ ^11^, she presented with hyperglycemia, hypernatremia, hypermagnesemia, mixed acidosis using the Stewart-Fencl-Figge method^12^, and neutrophilic leukocytosis (Tables 1, 2, 3 and 4).

Fluid resuscitation was carried out with 40 mL/kg, however, without improvement in circulatory shock signs. Inotropic support with epinephrine 0.05 mcg/kg/min was an attempt, with progressive titration up to 0.3 mcg/kg/min, however, without response. Then, the choice was therapy with norepinephrine (vasopressor), with initial dose of 0.05 mcg/kg/min until 0.5 mcg/kg/min, with pressure levels in the lowest limit (BP: 50×35 mmHg). Calcium correction was performed, and free water correction was maintained for the reduction of 8 mmol/L in 24-hour natremia, in association with basal electrolytes. It was, then, associated with hydrocortisone, in a shock dose of 100 mg/m^2^/day. The patient presented improved HR, peripheral capillary perfusion (PCP) and SBP; however, she presented with oligoanuria. Then, the choice was for expansion with physiological saline 20 mL/kg, red blood cell concentrate (10 mL/kg), and continuous furosemide, with initial dose of 3 3 mg/kg/day, with no response, and indication for peritoneal dialysis. Functional echocardiography showed distensibility index of inferior vena cava (dIVC) of 8%, using the Feissel method, global diffuse hypocontractility, without suggestive reinforcements of endocarditis, and mild pericardial effusion (<2 mm). The patient then evolved to refractory shock, without any response to the therapeutic measures available in the service, thus leading to death two days after pediatric intensive support.

## DISCUSSION

This case report emphasizes the fatal clinical course of an infant admitted with infection by SARS-CoV-2, associated with significant comorbidity, presenting with hyperinflammatory and multiple organ dysfunction syndromes. The clinical form presented in this report was myocarditis with elevated troponin levels, vasoplegic shock, continuous fever, cytopenia, hyperferritinemia, pulmonary involvement with mild to moderate ARDS^13^ and coagulopathy with hypofibrinogenemia and high D-Dimer levels, suggestive of a clinical condition that is similar to toxic shock syndrome.

The assumption is that the SARS-CoV-2 infection evolves in three stages,^14^ ^,^ ^15^ causing higher mortality rates in the third stage, two weeks or more after the onset of the symptoms. A minority of patients will reach the third stage, of the inflammatory cytokine storm, in which high levels of C-reactive proteins (CRP), ferritin, D-dimer, troponin and pro b-type natriuretic peptide can be detected, corroborating the diagnosis of inflammatory syndrome. The involvement of the myocardium in this condition is shown by very high cardiac enzyme levels during the course of the disease.

The clinical course of the patient in question was very acute, reaching the hyperinflammation stage in only two to three days after the onset of symptoms. Previous lung problems caused by extreme prematurity and bronchopulmonary dysplasia may have contributed with a low reserve, which led to the fatal outcome. However, this patient did not present with the severe form of ARDS, according to the current diagnostic criteria,^13^ nor had the initial need for elevated ventilatory parameters. The main changes associated with the worst clinical outcome were a result of cardiac involvement, vasoplegic shock and increased inflammatory markers, progressing to multiple organ dysfunction.

The clinical manifestations of this case report were similar to those described in other studies,^4^ ^,^ ^6^ ^,^ ^8^ with persistent high fever (38-40°C), significant gastrointestinal symptoms, evolving to warm, vasoplegic shock, refractory to volemic resuscitation and requiring noradrenaline and inotropic agents, as well as immune modulation therapies and the use of high doses of corticosteroids.

As in this case, most reports of MIS-C do not present significant respiratory involvement,^16^ ^,^ ^17^ ^,^ ^18^ using MV for cardiovascular stability. Other remarkable characteristics, besides persistent fever, myocardial injury and exacerbated inflammatory activity included the development of minor pleural, pericardial and ascitic effusions, which are suggestive of diffuse inflammatory process, as shown in this report.^19^ ^,^ ^20^ ^,^ ^21^

Even though the patient presented with a critical form of COVID-19, with laboratory evidence of infection or inflammation, including elevated concentrations of CRP, ferritin, triglycerides and D-dimers, no other pathological microorganism was identified.

It is important to mention that this patient was contaminated in the hospital environment, once she had been hospitalized since birth. It may have occurred after contact with her mother, after being transferred from the neonatal unit to the pediatric nursery, where a caretaker was required. The mother did not present with symptoms of COVID-19, which reinforces the importance of contamination surveillance in the hospital environment, considering the shared rooms in the pediatric nursery and asymptomatic individuals.

There is little information on the infection by SARS-CoV-2 in children with subjacent conditions and urgent need to collet standardized data that describe clinical presentation, severity, results and epidemiology of MIS-C in different regions, especially in scenarios where resources are limited, such as the Brazilian Amazon, and in the hospital environment, mainly among groups with higher risk for an erratic immune response.

Until the present moment, there have not been reports of cases suggestive of MIS-C associated with infection by SARS-CoV-2 in locations of limited resources, and in infants with history of extreme prematurity. This report reinforces the recommendations^14^ that all patients with the severe and/or critical form of COVID-19 be screened as to the presence of hyperinflammation, using laboratory biomarkers (for instance, ferritin dosage, CRP, blood sedimentation rate, (BSR) among others), and identifying the subgroup of patients for whom immunosuppression may increase the chances of mortality.
